# Bony avulsion of the supraspinatus origin from the scapular spine

**DOI:** 10.1007/s10195-011-0173-8

**Published:** 2011-12-02

**Authors:** Anne J. Vochteloo, Marjolijn Henket, Patrice W. Vincken, Jochem Nagels

**Affiliations:** 1 Department of Orthopaedics, Leiden University Medical Centre (LUMC), PO box 9600, 2300 RC Leiden, The Netherlands; 2 Department of Radiology, Leiden University Medical Center, Leiden, The Netherlands

**Keywords:** Avulsion, Scapular spine, Shoulder, Trauma, Supraspinatus, Conservative

## Abstract

We describe a case of an avulsion of the scapular spine at the origin of the supraspinatus muscle, with successful conservative treatment. An isolated avulsion is rare, as most avulsions occur in combination with other (more severe) injuries such as fractures of the scapula body or neck, coracoid process, glenoid or humerus. These injuries are mostly seen in high-energy trauma cases and need their own specific treatment. One should therefore always rule out concurrent trauma before treating conservatively.

## Introduction

The scapula is ossified from seven or more nuclei: one each for the body, the vertebral border and the inferior angle, and two each for the coracoid process and the acromion. The nuclei are fragile sites, susceptible to avulsions. Bony avulsions of different origins about the shoulder are described extensively in the literature, especially for children and adolescents. In most cases, they can be treated conservatively. In the case described in this report, we found an avulsion of the posterior–superior part of the scapular spine at the area of attachment of the supraspinatus (SS) and omohyoid muscles.

A PubMed search for this type of avulsion showed that in general they are found in combination with other lesions of the scapula or humerus [1–11].

To our knowledge, this is the first report of a case with an isolated avulsion of the origin of the SS tendon of the scapular spine.

## Case report

The patient was a healthy 19-year-old man who suffered a fall directly on his left shoulder during skiing. He was transferred to the local hospital and examined. The written report by the local doctor mentioned “a very painful shoulder, weakness of rotator cuff (RC) and normal neurovascular status”. No fractures or abnormalities were reported on the AP radiograph of the affected shoulder. Oral analgesics were prescribed, the arm was immobilized in a sling, and it was advised that an MRI to evaluate the RC should be obtained.

**Fig. 1:**
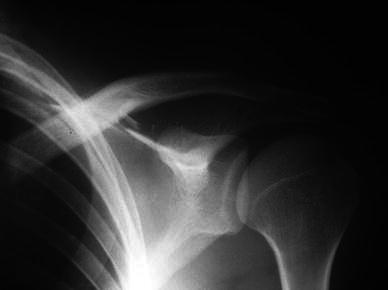
X-ray of the shoulder showing an avulsion of the scapular spine

**Fig. 2:**
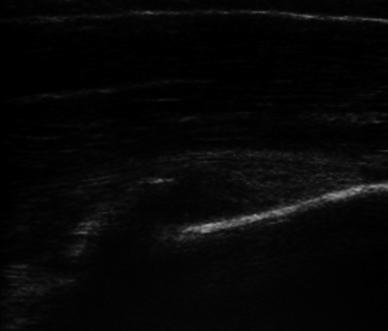
Ultrasound of the shoulder, showing a bony fragment within the muscle belly of the supraspinatus that is still attached to muscle fibers, and an interrupted cortical line of the scapular spine

We saw the patient 10 days later, still with a painful shoulder, but this had diminished substantially. On physical examination, there was tenderness over the scapular spine, no pain on palpation of the scapular body spine, and a full passive range of motion (ROM). The active ROM was limited to 80° of forward flexion and abduction due to pain. Active internal and external rotation was not impaired. There were no signs of instability. When evaluating the RC, a discrete weakness of the SS was found, which was painful when supplying counterforce to resistance. No neurological deficits were found. The initial X-rays were re-examined; a bony avulsion of the scapular spine was seen (Fig. 1). Additional X-rays of the scapula and shoulder revealed no further abnormalities. To evaluate the RC, an ultrasound was performed by an experienced radiologist (PV, Fig. 2). The bony fragment was identified as lying within the contours of the muscle belly of the SS, still attached to muscle fibres. The cortical line of the spine was interrupted, representing the site of the avulsion. The SS itself was intact and no other abnormalities were visualized. Based on these findings, we decided to treat this lesion conservatively.

Assisted active elevation and circumduction of the shoulder was initiated, followed by unrestricted active training at 6 weeks. At 2 months follow-up, the patient had full active ROM of his shoulder, including elevation and internal rotation. The strength of the SS was restored to normal. He was able to perform all activities without limitations. The patient gave informed consent prior to being included in this case report.

## Discussion

In general, a young patient will dislocate their shoulder or suffer a fracture of the greater tuberosity due to the force of the SS tendon when they fall on their shoulder. An avulsion on the scapular side, as seen in the present case, is rare. Most avulsions about the shoulder joint can be treated conservatively, as we did successfully. A review of the literature (available through PubMed) revealed several studies on avulsions of the scapula [1–12].

Most of the reported scapular avulsions occurred due to high-energy trauma and in combination with other fractures. In the largest series of scapula fractures, the fractures of the scapular spine were transverse (true) and not avulsions, and were due to high-energy trauma [1, 2].

Four studies have attempted to identify or to discuss the mechanism of avulsions [3–5, 12]. Heyse-Moore et al. [3] were not able to describe a specific mechanism for avulsions of the superior border of the scapula. However, their 7 cases did not involve a scapular spine avulsion. Avulsions of the spine of the scapula can be due to sudden forceful contraction of the SS or the omohyoid muscle; the latter was reported in 3 papers [5, 11, 12]. However, none of these papers mention avulsions due to SS force, as in our case.

All articles found were unanimous on the type of treatment applied: conservative [1–12]. Other concomitant fractures, mostly of the glenoid, scapula blade or neck, were often treated surgically [1, 6–10]. Wilber et al. [6] presented 40 scapula fracture cases and a literature review. Among their cases were just 3 spine avulsions (without any specific description), which were treated conservatively with good outcomes. In their series of conservatively treated scapular fractures with long-term follow-up, Schofer et al. [7] noted 3 spine avulsions (again, without specifying the exact location) with good outcomes after conservative treatment. Ogawa et al. [8] reported a larger series of superior border avulsions (24 cases: 19 length fractures, 5 short transverse), among which 1 had a concurrent avulsion of the scapular spine, treated conservatively.

It is interesting to see that in all of their cases, concomitant fractures of the shoulder were found, often of the coracoid process [8]. Two other papers reported on combinations of avulsions of the scapular spine and the AC joint or clavicle. One reported on 5 superior border avulsions of the scapula, among which 4 cases were combined with an AC dislocation [9]. The other 2 cases described suffereded from an avulsion of the cranial margin of the scapula, with either an AC dislocation or a lateral clavicle fracture [10].

With regards to the X-rays (reported to be normal) performed and the advice to have an MRI scan as part of further work-up, it is clear that careful review of the plain X-rays coupled with an inexpensive ultrasound confirmed the diagnosis in our case. However, the results and reliability of an ultrasound remain dependent on the experience of the radiologist. Complete X-ray series to evaluate the scapula contain an AP, a true lateral, an axillary and a Y view. If there is clinically obvious weakness of the RC, an MRI should be requested to rule out other, rarer pathologies, such as that presented by Tomas et al. [13].

This case emphasized that a thorough assessment of X-rays remains an important part of orthopaedic practice, and can definitely reduce costs.
